# Conservation of ethanol fermentation and its regulation in land plants

**DOI:** 10.1093/jxb/erz052

**Published:** 2019-02-28

**Authors:** Liem T Bui, Giacomo Novi, Lara Lombardi, Cristina Iannuzzi, Jacopo Rossi, Antonietta Santaniello, Anna Mensuali, Françoise Corbineau, Beatrice Giuntoli, Pierdomenico Perata, Mirko Zaffagnini, Francesco Licausi

**Affiliations:** 1 Institute of Life Sciences, Scuola Superiore Sant’Anna, Pisa, Italy; 2 Biology Department, University of Pisa, Pisa, Italy; 3 Department of Pharmacy and Biotechnology, University of Bologna, Bologna, Italy; 4 Global R&D, Valagro SpA, Atessa, Italy; 5 UMR 7622 CNRS-UPMC, Biologie du développement, Institut de Biologie Paris Seine, Sorbonne Université, Paris, France

**Keywords:** Alcohol dehydrogenase, anaerobic metabolism, anoxia, fermentation, hypoxia, plant evolution, submergence

## Abstract

Ethanol fermentation is considered as one of the main metabolic adaptations to ensure energy production in higher plants under anaerobic conditions. Following this pathway, pyruvate is decarboxylated and reduced to ethanol with the concomitant oxidation of NADH to NAD^+^. Despite its acknowledgement as an essential metabolic strategy, the conservation of this pathway and its regulation throughout plant evolution have not been assessed so far. To address this question, we compared ethanol fermentation in species representing subsequent steps in plant evolution and related it to the structural features and transcriptional regulation of the two enzymes involved: pyruvate decarboxylase (PDC) and alcohol dehydrogenase (ADH). We observed that, despite the conserved ability to produce ethanol upon hypoxia in distant phyla, transcriptional regulation of the enzymes involved is not conserved in ancient plant lineages, whose ADH homologues do not share structural features distinctive for acetaldehyde/ethanol-processing enzymes. Moreover, Arabidopsis mutants devoid of ADH expression exhibited enhanced PDC activity and retained substantial ethanol production under hypoxic conditions. Therefore, we concluded that, whereas ethanol production is a highly conserved adaptation to low oxygen, its catalysis and regulation in land plants probably involve components that will be identified in the future.

## Teaser

Transcriptional and biochemical comparisons in different phyla suggest the existence of alternative strategies of ethanol fermentation and its regulation in land plants.

## Introduction

Being aerobic organisms, plants rely on oxygen as the final electron acceptor in the mitochondrial electron transport chain dedicated to energy production. Its availability allows continuous flux of pyruvate, produced via the glycolytic pathway, into the tricarboxylic acid (TCA) cycle and the consequent electron transfer into the mitochondrial intermembrane space, where they return to the matrix via the F_O_F_I_–ATP synthase (Fernie *et al.*, 2004). Instead, when the oxygen concentration falls below the affinity of terminal oxidases, the electron movement is interrupted with an accumulation of TCA intermediates such as succinate and a drop in ATP production (António *et al.*, 2016). In such conditions, minimal energy production is maintained by coupling glycolysis with ancillary reactions that prevent the organism from arresting glycolysis due to NAD^+^ shortage. For instance, oxaloacetate reduction to malate by malate dehydrogenase by inverting the TCA cycle directionality at this step (Rocha *et al.*, 2010) or nitrate reduction via the cyclic action of nitrate reductases, nitrite reductases, and Phytoglobin1 have also been proposed to contribute to NAD(P)^+^ regeneration (Igamberdiev and Hill, 2004).

Common to several kingdoms of life is also the anaerobic activation of fermentative pathways. These reactions constitute an efficient solution to restore the pool of oxidized NAD^+^ while, at the same time, avoiding pyruvate and succinate accumulation. Indeed, these metabolites have been proposed to stimulate respiratory rates and, thereby, rapidly deplete the already scarce oxygen pool to nearly anoxic conditions (Zabalza *et al.*, 2009). Two fermentative pathways are expected to contribute mainly to glycolysis maintenance in plants: lactate and ethanol fermentation (Fukao and Bailey-Serres, 2004). The first is rapidly activated as a consequence of pyruvate dehydrogenase (PDH; EC 1.2.4.1) inhibition under oxygen deprivation and includes a single reaction to reduce pyruvate to lactic acid via lactate dehydrogenase (LDH; EC 1.1.1.27) (Perata and Alpi, 1993). Being a weak acid, lactic acid rapidly dissociates, contributing to the cytoplasmic acidification, which is initiated by the inactivation of vacuolar H^+^ ATPases (Gibbs and Greenway, 2003). The drop in cytoplasmic pH, in turn, inhibits LDH activity and stimulates that of pyruvate decarboxylase (PDC; 4.1.1.1), the first enzyme involved in ethanol fermentation (O’Carra and Mulcahy, 1997). Based on sequence similarity to yeast and bacterial proteins, plant PDC has been proposed to act as a thiamine-pyrophosphate-bound tetramer to catalyse the decarboxylation of pyruvate to acetaldehyde (Arjunan *et al.*, 1996; Kürsteiner, 2003). In Arabidopsis, four PDC-coding genes have been identified, two of which are hypoxia inducible (Mithran *et al.*, 2014).

Due to its high reactivity with nucleophilic compounds such as amino acids, acetaldehyde is expected to be rapidly reduced to ethanol using NADH as an electron donor (Dyda *et al.*, 1993). This last step is catalysed by plant-specific medium-chain alcohol dehydrogenases (ADH-P; EC 1.1.1.1), which are present in multiple copies in several species (Strommer, 2011). The active form of these enzymes consists of a homodimer in which two zinc ions are bound to each subunit. One zinc is co-ordinated by three Cys residues and the substrate, acting as a catalytic ion, whereas the second zinc atom is required for the stability of the protein structure and therefore, together with its four co-ordinating Cys, is referred to as the structural zinc site (Chen *et al.*, 2015). The activity of ADH is considered essential to enable tolerance to low oxygen conditions, since it serves the double purpose of detoxifying toxic acetaldehyde and regenerating oxidized NAD^+^ (Fukao and Bailey-Serres, 2004). The same enzyme has also been proposed to catalyse the reverse reaction, once aerobic conditions are restored, to recover carbon from ethanol and convert it into acetic acid via aldehyde dehydrogenase (AlDH; 1.2.1.3) (Pavelic *et al.*, 2000).

Several analyses using transgenic plants with altered ADH or PDC activity have demonstrated the importance of fermentation under low oxygen conditions in rice (Matsumura *et al.*, 1998; Saika *et al.*, 2006), maize (Johnson *et al.*, 1994), and Arabidopsis (Shiao *et al.*, 2002; Mithran *et al.*, 2014; Zhang *et al.*, 2016), with the latter enzyme acting as the rate-limiting step (Ismond, 2003).

Transcriptional up-regulation of PDC and ADH upon hypoxia has been reported in several angiosperm species. In Arabidopsis and barley, these two enzymes have been shown to be transcriptionally activated and sustained by ERF-VII proteins (Hinz *et al.*, 2010; Licausi *et al.*, 2010; Bui *et al.*, 2015), a group of ethylene response transcription factors whose localization, abundance, and activity are modulated in an oxygen-dependent manner (Gibbs *et al.*, 2011; Licausi *et al.*, 2011; Weits *et al.*, 2014). Indeed, a Hypoxia Responsive Promoter Element (HRPE), which is specifically recognized by group VII of ERF transcription factors, has been found in the promoter of these fermentative genes in different plant species (Gasch *et al.*, 2016). A trihelix transcription factor named Hypoxia Responsive Attenuator 1 is instead responsible for tuning down the induction of fermentative genes, by repressing ERF-VII activity (Giuntoli *et al.*, 2014).

Induction of ethanol fermentation is not exclusively actuated in response to low oxygen conditions. For instance, in Arabidopsis, co-ordinated expression of both PDC and ADH has been reported under cold, drought, and salt stress (Geng *et al.*, 2013; Song *et al.*, 2016; Kim *et al.*, 2017). Ethanol production in response to low temperature has been recently proposed to fluidize membranes and preserve their function under freeze–thaw stress, whereas its role under drought and salt conditions may also be explained by its activity as a signal to induce reactive oxygen species- (ROS-) detoxifying enzymes, a role supported by the application of exogenous ethanol (Kaplan *et al.*, 2009). Additionally, the concerted activity of PDC and ADH has also been observed in aerobic pollen grains. However, in these tissues, PDC channels pyruvate towards acetate production via AlDH, in a pathway defined as the ‘PHD bypass’, while ADH is only expected to buffer the system and prevent acetaldehyde toxicity (Bucher *et al.*, 1995; Mellema *et al.*, 2002; Strommer, 2011).

In Arabidopsis, only one protein is believed to account for acetaldehyde/ethanol interconversion, namely ADH1 (At1g77120) (Strommer, 2011). Plant genomes contain two additional classes of enzymes annotated as class III ADHs (EC 1.2.1.1), able to carry out glutathione-dependent oxidation of formaldehyde (Fliegmann and Sandermann, 1997), and cinnamyl alcohol dehydrogenases (CAD; EC 1.1.1.195), involved in the reduction of aromatic aldehydes (Kim *et al.*, 2004). In Arabidopsis, the only class III ADH, named ADH2, is dedicated to the reduction of nitrosylated glutathione (GSNO) and thereby is also referred to as nitrosoglutathione reductase (GSNOR) (Xu *et al.*, 2013; Zaffagnini *et al.*, 2016), whereas several CAD genes have been characterized to be involved in the production of monolignols for lignin biosynthesis and defence-related functions (Halpin *et al.*, 1994).

In the present study, we assessed the conservation of ethanol fermentation in land plants from an evolutionary perspective, comparing species belonging to phyla that represent progressive acquisition of distinctive traits from bryophytes to angiosperms. Our results show that, whereas this metabolic pathway seems to be maintained throughout plant evolution, the transcriptional regulation imposed on the genes that code for fermentative enzymes is a rather recent acquisition of seed plants. Taking advantage of Arabidopsis mutants impaired in fermentative metabolism, we observed that acetaldehyde production, rather than its consumption via ADH, is required to promote survival under low oxygen conditions. Surprisingly, we could still observe ethanol production in plants devoid of ADH1 expression and, in agreement with this, the most basal lineage of land plants, *Marchantia polymorpha*, does not appear to possess an ethanol-processing ADH. Altogether, our observation challenges the paradigm of ethanol production relying solely on ADH-like enzymes and their essential role in anaerobic glycolysis.

## Materials and methods

### Plant material and growth conditions

*Arabidopsis thaliana* Col-0 (Columbia-0) ecotype was used as the wild type in all experiments. The T-DNA insertion mutants *adh1* (N552699) and *adh2* (N430191) were obtained from the Nottingham Arabidopsis Stock Centre (NASC), and the *pdc1pdc2* double mutant was obtained by crossing N660027 and N862662 (*pdc1* and *pdc2* mutant, respectively, described by Mithran *et al.*, 2014). Homozygous lines were identified via PCR screening of genomic DNA using gene-specific primers together with T-DNA-specific primers. Double homozygous lines were obtained by crossing the two single mutants and then screening the F_2_ generation as described above.

*Pinus pinea* nuts were collected along the Arno river in a 300 m^2^ area centred around Google maps co-ordinates 43.704733 and 10.424682. *Pteris vittata* spores were collected from spontaneous plants found in the garden surrounding Casa Pacini of the Department of Crop Plants of the University of Pisa (co-ordinates 43.704733 and 10.424682). *Selaginella moellendorfii* was purchased from Bowden Hostas and propagated vegetatively. *Physcomitrella patens* was provided by Tomas Morosinotto (University of Padova). *Marchantia polymorpha* Cam2 was provided by Linda Silvestri (University of Cambridge).

Arabidopsis plants were grown in a soil consisting of peat:perlite at a ratio of 3:1. Seeds were stratified at 4 °C in the dark for 3 d and germinated in a growth chamber under temperatures of 23 °C/18 °C (day/night) and a light cycle of 12 h/12 h (light/dark) with 80–120 µmol photons m^−2^ s^−1^ irradiance. *Pinus pinea*, *P. vittata*, and *S. moellendorffii* were grown on perlite soil under growth chamber conditions as described above for *A. thaliana*. *Physcomitrella patens* was cultured in sterile conditions on solid Knop medium described in Reski and Abel (1985) while *M. polymorpha* was cultured in solid half-strength Murashige and Skoog (MS) medium (0.9% w/v agar).

For experiments in axenic culture using 6-well plates, Arabidopsis seeds were surface sterilized with 70% ethanol (v/v) followed by 0.4% NaClO (v/v) and rinsed six times with sterile distilled water. Seeds were sown in liquid half-strength MS medium (pH 5.8) supplemented with 1% (w/v) sucrose, then stratified at 4 °C in the dark for 3 d and transferred to growth chamber conditions (as described above).

*Pinus pinea*, *P. vittata*, and *S. moellendorffii* were grown on perlite soil under growth chamber conditions as described above. Explants were surface sterilized following the same protocol used for Arabidopsis seeds, and transferred to liquid medium for 2 d before being subjected to further experiments. *Physcomitrella patens* was cultured in solid Knop medium described in Reski and Abel (1985) whereas *M. polymorpha* was cultured in solid half-strength MS medium. Agar was used at 0.8% (w/v) to solidify the media. Both species were grown with a temperature regime of 23 °C and 18 °C day/night and a 12 h photoperiod with 80–120 µmol photons m^−2^ s^−1^ irradiance.

Hypoxic treatments were applied in the dark to avoid oxygen release by photosynthesis. Pre-mixed gas bottles containing 99% N_2_ and 1% O_2_ (v/v) were used to provide the desired hypoxic atmosphere to the plants, in plexiglas boxes. Aerobic controls were obtained by flushing compressed air in a similar way.

### Construct design and plant transformation

The full-length coding sequence of *MpADH1* (Mapoly0154s0033) was amplified from *M. polymorpha* cDNA using Phusion High Fidelity DNA polymerase (Thermo-Fisher Scientific) using gwMpADHf (caccATGGGAAGCGCCTCGGATGA) and gwMpADHr (CTAGTCCCAGCACGAGATGACTG) primers. and cloned into pENTR-D TOPO vector (Thermo-Fisher Scientific) to obtain the entry vector pENTR-MpADH. This was recombined into the destination vector pH7WG2 (Karimi *et al.*, 2002) using the GATEWAY LR clonase II enzyme mix (Thermo-Fisher Scientific) to generate the expression vector 35S:MpADH. This construct was stably introduced into the *adh1* mutant background of Arabidopsis, using the floral dip method (Zhang *et al.*, 2006). T0 seeds were screened for hygromycin resistance, and transgene lines were identified using standard PCR with a gene-specific forward primer and attB2 reverse primer, or real-time quantitative PCR on genomic DNA with gene-specific primers, and single-copy insertions were confirmed by segregation analysis. Homozygous T_3_ or subsequent generations were used in the following experiments.

### Anoxia tolerance and hypoxic germination assays

One-week-old Arabidopsis plants grown on vertical plates were subjected to an anoxic atmosphere for the duration indicated in the figure legends, inside a glovebox chamber (Anaerobic System model 1025; Forma Scientific). All treatments started at the end of the day (ZT12). Tolerance was assessed 7 d after the end of the treatment by scoring plants as alive (no visible damage), stunted (partial leaf bleaching), or dead (complete bleaching and arrest of growth) and comparing the proportion of observations in these categories using a χ^2^ test. Seeds were incubated for 4 d at 4 °C in darkness in order to break seed dormancy and then placed at 22 °C under continuous light in atmospheres containing 1, 3, 5, 10, and 21 % (v/v) oxygen. Oxygen tensions were achieved through mixing N_2_ and air via capillary tubes according to the device described by Côme and Tissaoui (1968). Germination percentage was scored after 7 d.

### Real-time quantitative RT–PCR analysis

Total RNA was extracted from plant tissues using the Spectrum Plant Total RNA kit (Sigma Aldrich), and subjected to DNase treatment using the RQ1-DNase kit (Promega) according to the manufacturer’s instructions. A 3 μg aliquot of RNA was reverse transcribed into cDNA using the iScript™ cDNA Synthesis Kit (Biorad). Real-time PCR amplification was carried out with the ABI Prism 7300 sequence detection system (Applied Biosystems), using aiQSYBR Green Supermix (Biorad). The primers used in qPCR were designed using the Quantprime software (Arvidsson *et al.*, 2008) listed in Supplementary Table S1 at *JXB* online, Relative quantification of the expression of each individual gene was performed using the 2^(–ΔΔC_t_) method (Livak and Schmittgen, 2001) using one (*A. thaliana*, *P. pinea*, and *P. patens*) or the geometric average of three housekeeping genes (*P. vittata*, *S. moellendorffii*, and *M. polymorpha*) as a normalization reference, also listed in Supplementary Table S1.

### Quantification of ethanol production

Plant tissues grown *in vitro* were incubated for 12 h with half-strength MS medium (pH 5.8) supplemented with 2% sucrose (w/v) to sustain ethanol production when subjected to the aerobic or hypoxic treatment indicated in the figure legends. At the end of the treatment, the residual liquid medium was collected and the ethanol release per milligram of fresh weight of plant material was measured as described by Licausi *et al.* (2010).

### Phylogenetic and protein structure analysis

Identification of ADH and PDC protein sequences in different sequenced plant species was performed by searching the phytozome database (www.phytozome.net). *Pteris vittata* and *P. pinea* sequences were retrieved from the 1000 Plants transcriptome database (www.onekp.com) and EuropineDB (http://www.scbi.uma.es/pindb/), respectively. Protein sequences similar to *A. thaliana* PDC1 (At4g33070), ADH1 (At1g77120), or ADH2 (At5g43940) were retrieved using the BLAST algorithm (Altschul *et al.*, 1990). An initial selection of protein sequences was based on a Blast score >400 and Expect-value <1e^-20^. Reciprocal blasting against the Arabidopsis proteome database was used to confirm their identity as closest orthologues of the Arabidopsis sequences initially used as bait. Phylogenetic analyses were performed using MEGA7 (Kumar *et al.*, 2016). To obtain the phylogenetic tree, ADH protein sequences from different species were aligned using the MUSCLE algorithm (Edgar, 2004). The maximum likelihood method was used to construct the tree. To test the reliability of the tree, we used the bootstrapping method with 500 replicates. Three-dimensional protein structures of Arabidopsis ADH1 (PDB ID: 4rqt) and *Equus caballus* liver ADH (PDB ID: 1adc) were retrieved from the protein Databank in Europe (https://www.ebi.ac.uk/pdbe/entry/pdb/4RQT), aligned, and structurally matched using UCSF Chimera (Pettersen *et al.*, 2004) to obtain a prediction of ethanol positioning within the substrate-binding pocket of ADH1.

### Protein extraction and enzyme activity assay

Total soluble proteins were prepared as described previously (Russell *et al.*, 1990) with minor modifications. Briefly, seedlings extracts (200 mg) were prepared by homogenization of material in ice-cold extraction buffer containing 50 mM Tris–HCl buffer (pH 7.5), 0.5 mM EDTA, 1 mM phenylmethylsulfonyl fluoride (PMSF), and 10 mM DTT. The extracts were then centrifuged (10 000 *g* for 15 min at 4 °C) to remove insoluble material. Protein amounts were measured using the Bradford method (Bradford, 1976) with BSA as a standard. Enzyme activity was determined at room temperature by incubating the protein extract (0.2 mg) in 1 ml of 0.1 M glycine-NaOH (pH 10) containing 0.2 mM NAD^+^ and 50 mM ethanol as described by Cheng *et al.* (2013). ADH activity was monitored for 5 min after the addition of ethanol using an Agilent Cary60 UV-Vis spectrophotometer. The rates of the reaction were corrected with a reference rate without ethanol. Rates were averaged over selected intervals during which the absorbance increase was linear. Final activity values were normalized against total protein amount. Data are means of four independent biological experiments.

### Immunoblotting

Arabidopsis seedlings where grown axenically in liquid MS medium supplemented with 1% sucrose (w/v), as described above. Total soluble proteins were extracted from 4-day-old whole seedlings, with a buffer composed of 50 mM Tris–HCl (pH 8), 1 mM EDTA (pH 8), 100 mM NaCl, 2% SDS, 0.05% Tween-20 (v/v), and protease inhibitor cocktail (Sigma-Aldrich) as recommended by the manufacturer. The plant material was ground in liquid nitrogen into a fine powder, quickly extracted, and spun at 8000 *g* for 10 min at 4 °C. After quantification with the Pierce BCA Protein Assay kit (Thermo-Fisher Scientific), 50 μg of total proteins from the cleared supernatant were denatured and separated by SDS–PAGE, using 10% Bis-Tris Novex gels (Thermo-Fisher Scientific). ADH immunodetection was performed by means of a polyclonal anti-ADH antibody (Agrisera, cat. no. AS10 685, working dilution 1/3000) and an anti-rabbit horseradish peroxidase (HRP)-conjugated secondary antibody (Agrisera, cat. no. AS09 602, working dilution 1/20 000). Luminescence was elicited by incubation of the membrane with the LiteAblot Turbo ECL Substrate (EuroClone) and imaged with a ChemiDoc system (Bio-Rad).

### Statistical analysis

Comparison between treatments was analysed by *t*-test or one-way ANOVA applying a Holm–Sidak post-hoc test depending on the number of averages compared. In case of failure of the Shapiro–Wilk test or normality of data distribution, an ANOVA on ranks was performed to compare more than two categories, followed by a Tukey’s post-hoc test for multiple comparisons, or Welch’s *t*-test in the case of two categories only. Comparison of anoxia tolerance in different Arabidopsis genotypes was evaluated by the proportion of observations in categories applying a χ^2^ test. Letters were used to distinguish groups that differ significantly, as indicated in the figure legends. Asterisks were used to indicate *P*-values (*P<0.05, **P<0.01,***P<0.001).

## Results

**Fig. 1.**
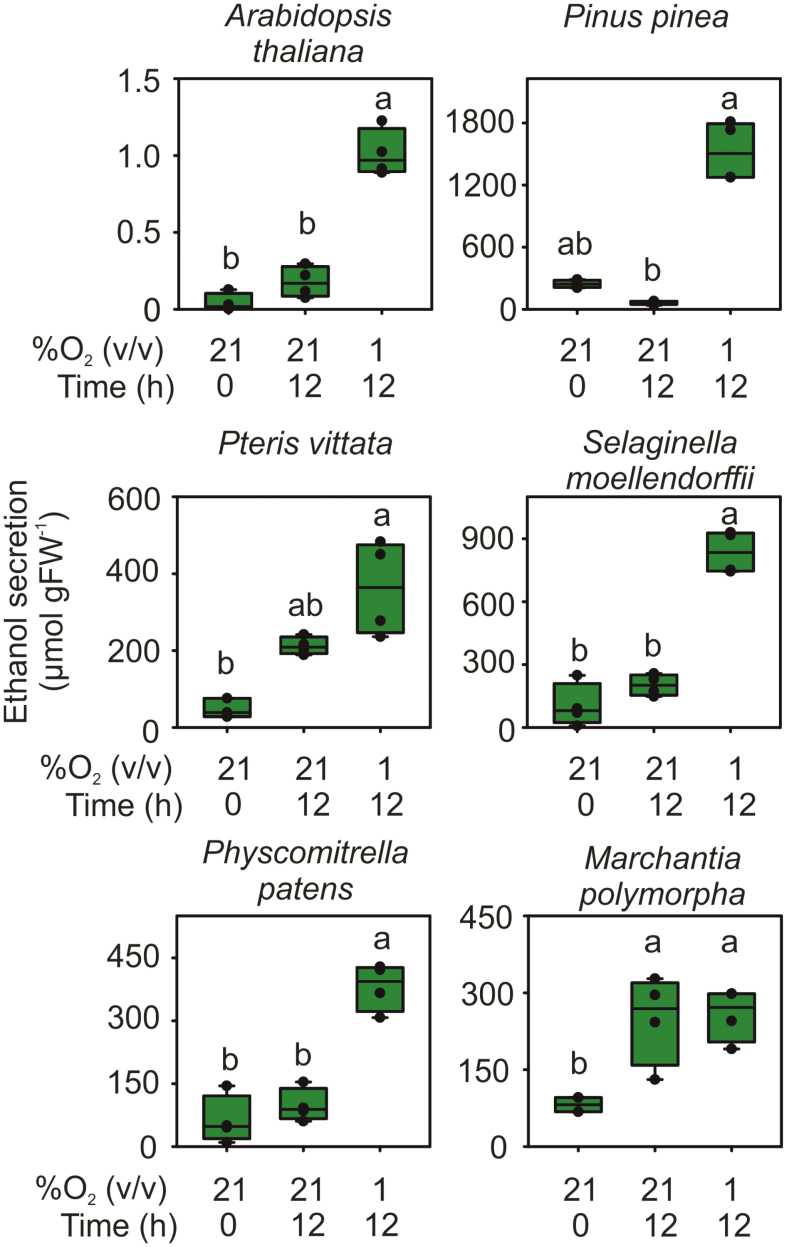
Ethanol production in tissues of species representing evolutionary steps in land plants from bryophytes to angiosperms. Samples were transferred to sterile media containing 2% (w/v) sucrose 12 h before (Time 0) being incubated for 12 h with an artificial atmosphere containing either 21% or 1% (v/v) oxygen. The box plots are built on the basis of four replicates. Letters indicate statistical differences between conditions (*P*<0.05). The tissues used were: 1-week-old seedlings for *A. thaliana*, 2-week-old seedlings for *P. pinea*, mature sporophyte for *P. vittata* and *S. moellendorffii*, mature gametophore for *P. patens*, and gametophyte for *M. polymorpha*.

To test whether the activation of ethanol fermentation is a conserved feature in the plant kingdom, we assessed ethanol production by species representing evolutionary steps towards the development/establishment of flowering plants. One plant species from each main plant phylum was chosen: *A. thaliana* for angiosperms, *P. pinea* for gymnosperms, *P. vittata* for pteridophytes, *S. moellendorffii* for lycophytes, *P. patens* for bryophytes, and *M. polymorpha* for epathophytes. Individuals belonging to each of these plant species were grown *in vitro* under sterile conditions to developmental stages that could be considered as juvenile or adult, and transferred in a 2% (w/v) sucrose medium for 12 h before the application of aerobic (21% O_2_ v/v) or anaerobic (1% O_2_ v/v) conditions for an additional 12 h. Medium samples were collected before or at the end of the treatment, and the amount of ethanol released was quantified by an enzymatic assay. This analysis revealed that, in these experimental conditions, all plant species released ethanol under hypoxic conditions (Fig. 1). *Arabidopsis thaliana* seedlings exhibited a very limited, although detectable, amount of ethanol release in the range of 1 nmol g^–1^ FW, whereas all other species accumulated tens to hundreds of times more ethyl alcohol in the medium. With the exception of *M. polymorpha* and *P. vittata*, all other species released more ethanol under anoxic conditions as compared with normoxia (Fig. 1), hinting at a conserved stimulation of this pathway by oxygen deficiency. The growth medium where the tested epathophyte was grown showed substantially equal ethanol amounts under aerobic and anaerobic conditions, although both were higher than the initial time point considered (Fig. 1), indicating a possible stimulatory effect of sucrose alone. The fern *P. vittata* exhibited a similar trend, with a significant increase in ethanol accumulation under normoxia, although this was further increased by hypoxia (Fig. 1). Considered together, these results indicate that stimulation of ethanol production by oxygen deficiency is a conserved feature among plant phyla, with the exception of epathophytes.

To understand whether plant phyla maintained transcriptional control over fermentative metabolism under oxygen deprivation, we carried out a time-resolved survey of the expression of different *ADH* and *PDC* genes, in the same species where ethanol production was previously assessed. First, we searched for putative orthologues of the Angiosperm PDC and ADH proteins in public proteome databases, using reciprocal blast against the Arabidopsis sequences ADH1 (At1g77120) and PDC1 (At4g33070). All land pant species tested possess at least one gene coding for a protein with sequence similarity to the Arabidopsis ADH and PDC enzymes (Supplementary Table S2).

Whereas putative PDC proteins could be unambiguously identified, prediction of ethanol dehydrogenases presented some challenges, due to the high similarity between enzymes that act on different substrates. Especially in the case of several ADH-like proteins identified in bryophytes and primitive vascular plants, reciprocal blasting could not help to distinguish between ADH1- and GSNOR-like sequences (Supplementary Table S2).

**Fig. 2.**
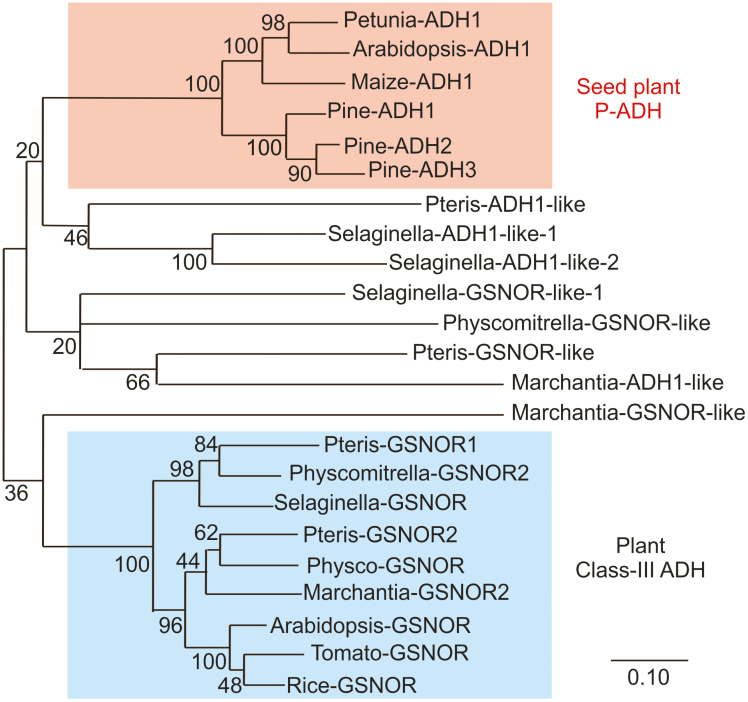
Phylogenetic tree illustrating the relatedness of ADH proteins identified in species representing land plant evolution (*Arabidopsis thaliana*, *Zea mays*, *Petunia hybrida*, *Solanum lycopersium*, *Pinus pinea*, *Pteris vittata*, *Selaginella moellendorffii*, *Physcomitrella patens*, and *Marchantia polymorpha*). MEGA7 (Kumar *et al.*, 2016) was used to generate an unrooted maximum-likelihood tree. The original alignment is shown in Supplementary Dataset S1. Red and blue shadings indicate significantly reproducible (bootstrap value >90) clustering of ADH1-like sequences and GSNOR-like sequences, respectively.

We therefore aligned the ADH-like sequences retrieved in this way, including well-characterized ADH1 or GSNOR sequences from angiosperms, and generated a phylogenetic tree representing their relatedness (Fig. 2; Supplementary Dataset S1). A separation in two main clades could be observed (Fig. 2), where ethanol dehydrogenases and GSNOR sequences clustered apart. However, while proteins from seed plants reliably clustered together within each of the two classes, a less defined grouping was achieved for sequences belonging to early vascular plants.

**Fig. 3.**
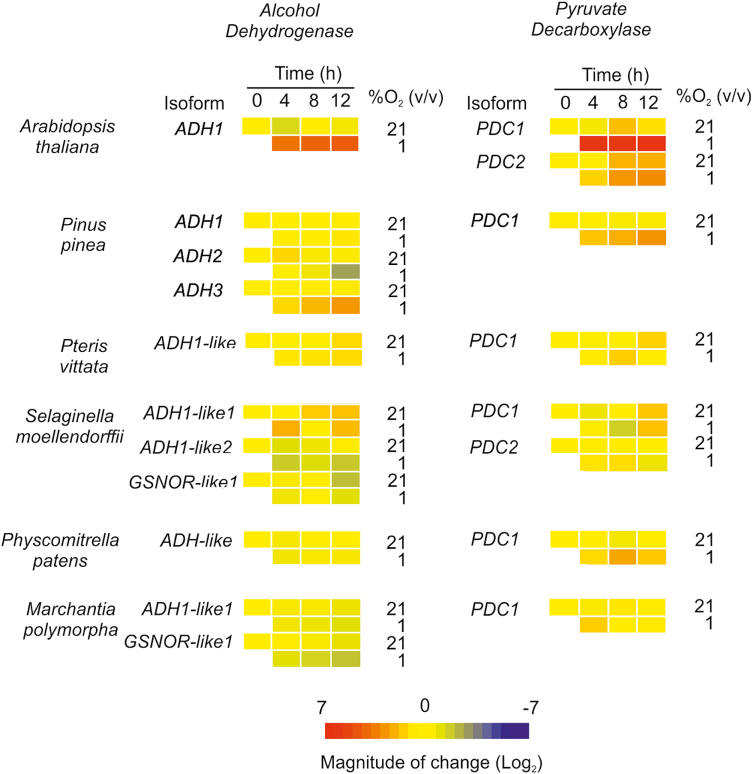
Expression analysis of genes coding for putative fermentative enzymes in response to hypoxia in different plant species that represent subsequent steps in the evolution of land plants. mRNA was extracted from samples treated with 0, 4, 8, and 12 h of hypoxia (1% O_2_ v/v) and the expression of ADH- and PDC-like genes was compared with that of plants maintained in normoxia. Numeric expression values are shown in Supplementary Table S2.

The expression of the genes encoding the putative PDC and ADH enzymes previously selected was monitored under aerobic and anoxic conditions over a 12 h time frame by sampling plant material every 4 h from the beginning of the day (ZT0). Transcriptional activation of both fermentative enzymes under anoxia was observed in Arabidopsis, where the mRNA levels of *ADH1* (*At1g77120*) and two *PDC* isoforms (*At4g33070* and *At5g54960*) were up-regulated as previously reported (Mithran *et al.*, 2014) (Fig. 3; Supplementary Table S3). Similarly, one ADH gene and one PDC gene were induced by anoxic conditions in pine at all time points considered. A less pronounced effect was instead observed in the remaining species: moderate induction of the identified *PDC* genes was observed, although a less clear pattern was evident for *Selaginella* and *Pteris* genes, whose expression throughout the 12 h time frame varied already under aerobic conditions (Fig. 3; Supplementary Table S3). *ADH* expression in these last two species was not enhanced by anoxia, when some isoforms instead exhibited down-regulation as compared with their aerobic controls. In summary, whereas a clear up-regulatory trend could be appreciated in spermatophytes, more ancient land plants showed species-specific and small-magnitude changes, mostly limited to PDC genes.

Since we observed a significant increase of ethanol production under hypoxic conditions (Fig. 1), we concluded that early land plants do not rely on transcriptional control of fermentative genes, and the activation of this pathway under oxygen deficiency might rather be achieved in these species by post-transcriptional activation of fermentative enzymes or simply by pyruvate accumulation.

**Fig. 4.**
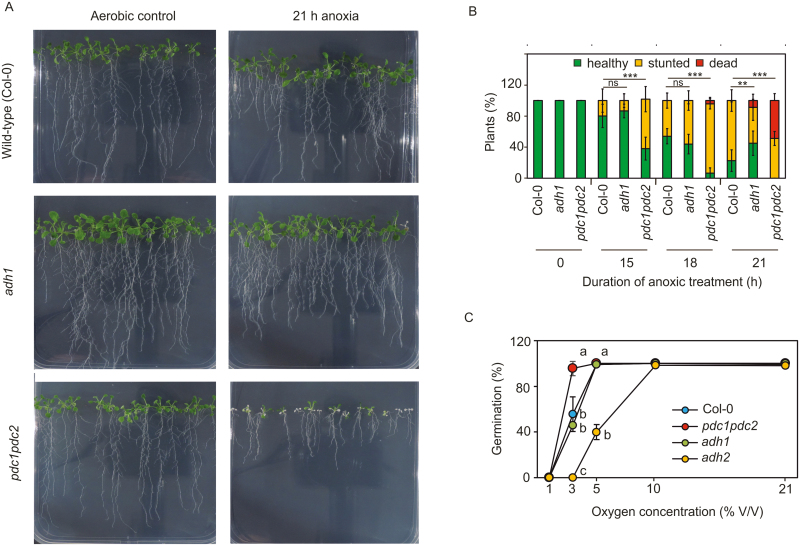
Contribution of hypoxia-inducible ADH and PDC genes to low oxygen conditions. (A) Phenotypic difference of plants treated with 21 h with anoxia. (B) Progressive scoring as arbitrary units of wild type (Col-0) plants and *adh1* or *pdc1pdc2* knock-out mutants subjected to an anoxic treatment of a variable duration. Comparison among different Arabidopsis genotypes was evaluated by the proportion of observations in categories applying a χ2 test. (C) Germination percentage of wild-type and *adh1*, *adh2*, and *pdc1pdc2* seeds under variable oxic atmospheres after seven days. Statistical significance of the differences observed between genotypes at each time point was assessed by 1-way ANOVA followed by Holm-Sidak post-hoc test (*n*=5).

Independently of its regulation, the conservation of ethanol fermentation among land plants suggests the essentiality of this pathway to cope with respiratory inhibition under anaerobiosis. Indeed, reduction to ethanol contributes to NAD^+^ regeneration to sustain glycolytic fluxes. Additionally, ADH activity might help in depleting toxic acetaldehyde pools that build up following pyruvate decarboxylation (Perata and Alpi, 1991). To our surprise, however, an Arabidopsis genotype devoid of the two main *PDC* genes that are induced by low oxygen conditions (*pdc1pdc2*) exhibited lower tolerance to complete anoxia than an *adh1* mutant, which instead was only slightly more sensitive than wild-type plants after 21 h treatment (Fig. 4A, B). This is in disagreement with the aforementioned hypothesis, since acetaldehyde is expected to accumulate and NAD^+^ to be regenerated exclusively by LDH in the *adh1* mutant. The double *pdc1pdc2* mutant also exhibited higher seed germination rates under hypoxic conditions than the wild type, which was not different from *adh1*, again hinting at a major role in pyruvate decarboxylation rather than alcohol/acetaldehyde reversible conversion.

In contrast to ADH1, the ADH2 isoform appeared to play a major role in seed germination under suboptimal oxygen conditions, as demonstrated by the low germination rate of its corresponding knock-out mutant at 3% and 5% O_2_ (v/v) (Fig. 3C).

**Fig. 5.**
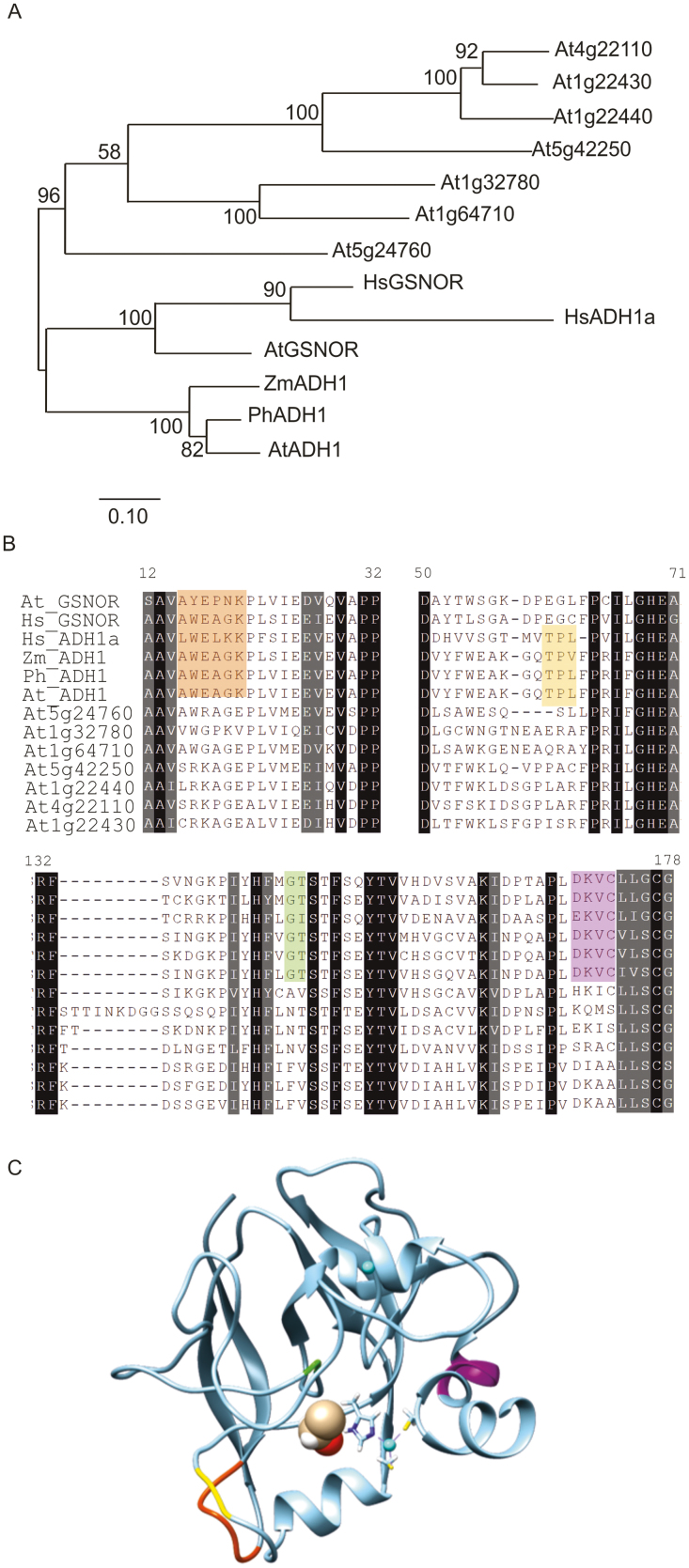
Analysis of structure and similarity within Arabidopsis ADH1 homologues. (A) A Phylogenetic tree depicting the relatedness of ethanol-processing dehydrogenases (At_ADH1, PhADH1, ZmADH1, and Hs_ADH1), GSNO reductases (At_GSNOR and Hs_GSNOR), and ADH-like sequences of unknown function (At5g24760, At1g64710, At1g32780, At5g42250, At5g42250, At1g22440, At1g22430, and At4g22110). Bootstrap confidence levels (*n*=500) are shown at each branching site. The complete alignment from which the tree derives is contained in Supplementary Dataset S2. (B) Multialignment of protein regions that contain ADH1/GSNOR distinguishing motifs (coloured shading). Black–grey shadings indicate conserved domains among all ADH proteins considered. (C) Three-dimensional representation of the substrate-binding domain (1–184) of the Arabidopsis ADH1 (PDB ID: 4rqt). Distinctive ADH1/GSNOR motifs are shown in the same colours used to highlight the sequences in (B). Ethanol (brown for carbon, white for hydrogen, and red for oxygen atoms) and zinc ions (pale green) are shown using spherical representations. The side chains of His and Cys residues involved in catalytic zinc co-ordination are shown.

The absence of substantial differences in plant performance under low oxygen between the wild type and *adh1* mutants (Fig. 4) led us to speculate whether additional enzymes in Arabidopsis are able to operate on acetaldehyde and ethanol as substrates. A BLAST (Altschul *et al.*, 1990) search using the ADH1 amino acid sequence against the Arabidopsis proteome returned 19 protein isoforms, corresponding to nine genes, with similarity score higher than HER2, an oxidoreductase involved in transducing the perception of E-2-hexenal (At5g63620) (Supplementary Table S4). We aligned the retrieved protein sequences in an attempt to identify those best resembling ethanol dehydrogenases in terms of conserved amino acid residues. To this end, we included in the list well-characterized ADH sequences from *Zea mays* and *Petunia×hybrida*, as well as the human ADH1a and GSNOR sequences (Supplementary Dataset S2). A phylogenetic tree representing the relatedness of these protein sequences is shown in Fig. 5A. Arabidopsis ADH1 and ADH2 consistently clustered together with their mammalian counterparts, whereas all other ADH-like proteins segregated apart. Manual inspection of the alignment led to the identification of three regions in the N-terminal part, which correspond to the catalytic and substrate-binding domain, able to distinguish ethanol dehydrogenases and GSNORs from the other Arabidopsis sequences (Fig. 5B). The conserved D_17_ and K_20_ (orange shading in Fig. 5B and loop in Fig. 5C) and sequence D_170_KVC_173_ (purple shading in Fig. 5B) localize in an external loop and helix, respectively, while a G_145_T_146_ loop (green shading in Fig. 5B) folds within the inner pocket in the proximity of the substrate. A T_60_P(L/V)_ 62_ sequence distinguished ethanol-processing sequences from all others considered (yellow shading in Fig. 5B). Additionally, differentiation of GSNOR from short-chain alcohol dehydrogenases was enabled on the basis of a S_67_G(K/A)DPEG_73_ sequence in the catalytic domain, and two H_298_K_299_ and T_318_RPFQLV_324_ motifs in the cofactor-binding domain (Supplementary Dataset S2). The alternative residues identified in the Arabidopsis ADH-like sequences suggested that these isoforms preferentially act on alternative alcohols and aldehydes. In conclusion, this sequence and structure analyses supported the hypothesis that a single ADH isoform is dedicated to ethanol/acetaldehyde conversion in Arabidopsis.

**Fig. 6.**
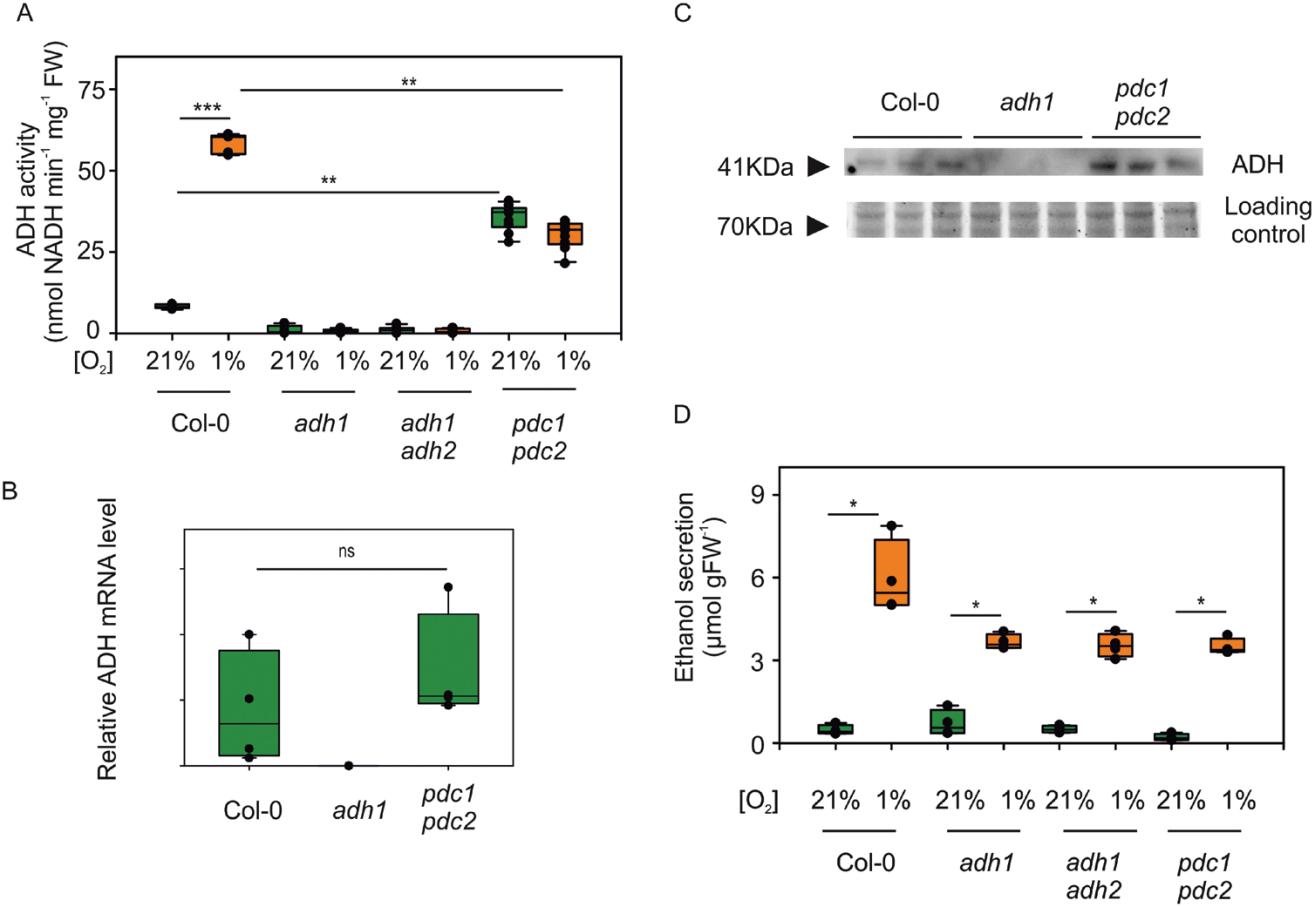
Assessment of ethanol fermentation in *ADH* and *PDC* mutants. (A) ADH activity in 6-day-old Arabidopsis seedlings (Col-0 wild type, and *adh1*, *adh1adh2*, and *pdc1pdc2* mutants) after incubation under normoxic (21% O_2_ v/v) or hypoxic (1% O_2_ v/v) conditions for 12 h. The box plots are built on the bases of four replicates. (B) *ADH1* expression in the wild type (Col-0), and *adh1* and *pdc1pdc2* mutants. The box plots are built on the bases of four replicates. (C) Immunodetection of ADH1 protein in aerobic wild-type (Col-0), *adh1*, and *pdc1pdc2* seedlings, grown in liquid MS medium for 4 d, in the presence of 1% sucrose (w/v). Three biological replicates for each genotype are shown. Gel staining with Coomassie G-250 is shown as a protein loading control (D) Ethanol production in the same genotypes used for the ADH activity quantification shown in (A). The box plots are built on the bases of four replicates.

To test this hypothesis experimentally, we compared ADH activity (hereafter referred to as ethanol to acetaldehyde conversion) and ethanol production in wild-type Arabidopsis seedlings, in the *adh1* mutant and the double *pdc1pdc2* knock-out genotype. *In vitro* NAD^+^-dependent ethanol oxidation in samples from aerobically grown and hypoxia-treated seedlings was almost entirely abolished in the *adh1* mutant, confirming that ADH1 is the only ethanol-processing dehydrogenase in Arabidopsis (Fig. 6A). ADH activity was induced ~5-fold by a 12 h hypoxic treatment in the wild type (Fig. 6A), in agreement with the increased expression at the mRNA level reported before (Fig. 3). Surprisingly, the ADH activity in the *pdc1pdc2* mutant under aerobic conditions reached levels significantly exceeding 65% of those observed in the anaerobic wild type, although it did not increase further under hypoxia, suggesting the existence of non-stress-related regulation of ADH activity (Fig. 6A). We therefore tested whether this regulation occurs at the transcriptional or translational level. Real-time quantitative PCR assessment of the *ADH1* mRNA levels in the double *pdc1pdc2* mutant did not show significant differences as compared with the wild type (Fig. 6B). Instead, immunoblotting using an anti-ADH antibody revealed that ADH1 protein was more abundant in the double mutant than in the wild type, whereas the *adh1* mutant, as expected, was completely devoid of it (Fig. 6C). Remarkably, when we quantified ethanol in the liquid medium where seedlings had been grown, we observed an accumulation in all hypoxic samples considered (Fig. 6D). Hypoxic *adh1*, *adh1adh2*, and *pdc1pdc2* seedlings leaked significantly more alcohol than their aerobic counterpart plants, in amounts within the quantification range of the enzymatic assay (Fig. 6D). A GC analysis confirmed the release of ethanol in the medium by *adh1* and wild-type seedlings under hypoxia (Supplementary Fig. S4). Given the high substrate specificity of ScADH for ethanol, we speculated that hypoxic conditions stimulated its production through a hypothetical pathway that does not involve PDC or ADH.

**Fig. 7.**
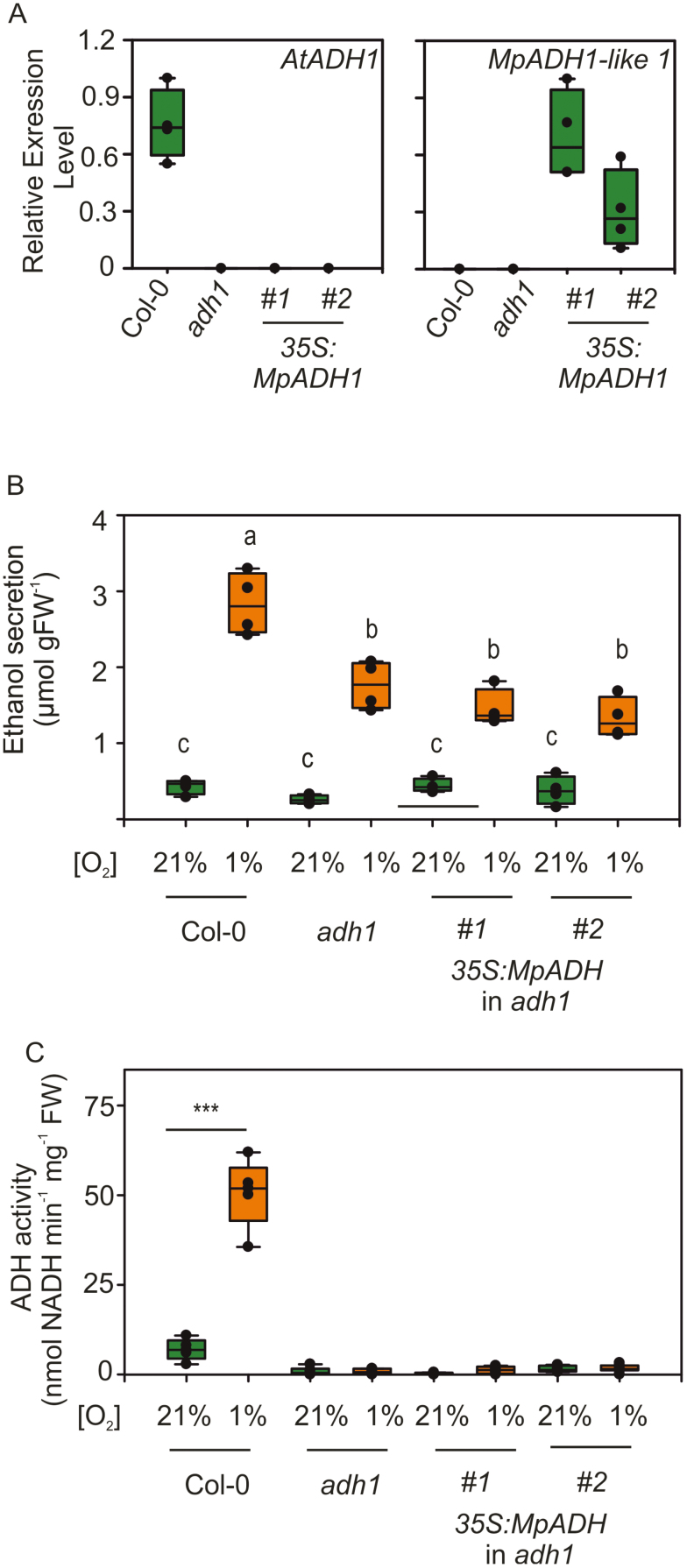
MpADH1 is unable to complement the absence of ADH1 in *A. thaliana*. (A) Ethanol production in wild-type, *adh1* mutant, and MpADH1-expressing *adh1* seedlings grown for 6 d and subsequently subjected to normoxic or hypoxic conditions for 12 h in the presence of 2% (w/v) sucrose (*n*=4). (B) Ethanol dehydrogenase activity measured in plant extracts of the genotypes analysed in (A) (*n*=4).

The observation of ethanol fermentation in the Arabidopsis *adh1* mutant led us to speculate whether the same alternative pathway to ethanol biosynthesis is also active in early tracheophytes and bryophytes. In the two representative species considered in the previous experiments, we could only identify sequences with limited homology to the ethanol dehydrogenases of angiosperms (Fig. 2; Supplementary Table S2). *Marchantia* ADH1-like protein, for instance, did not possess the four amino acid sequences identified in ethanol dehydrogenases (Fig. 5). To test whether these proteins are capable of aldehyde to ethanol conversion, we cloned the ADH1 coding sequence from *M. polymorpha* and expressed it under the control of the viral 35S promoter in the Arabidopsis *adh1* mutant (Fig. 7A). The resulting homozygous progenies were then tested for its ability to produce ethanol under either aerobic or hypoxic conditions. As observed before, the *adh1* plants included as control showed ethanol release in the medium under hypoxia, although lower than the wild type (Fig. 7B). No significant difference between the *adh1* mutant background and the *MpADH1* overexpressors was detected, in terms of ethanol secretion in control or hypoxic conditions (Fig. 7BA). In agreement with this last observation, expression of *MpADH1* could not compensate for the absence of the endogenous ADH1 in terms of ethanol to acetaldehyde interconversion, whereas the wild-type control exhibited a moderate activity under aerobic conditions, which increased following hypoxic treatment (Fig. 7C). We therefore concluded that the *Marchantia* enzyme with the highest similarity to the angiosperm ethanol dehydrogenases is unable, alone, to catalyse the acetaldehyde to ethanol conversion *in planta*, suggesting that also in its original milieu it is active on alternative substrates.

## Discussion

Ethanol fermentation has been shown to act as an ancillary reaction to glycolysis, which is constantly fuelling with oxidized NAD^+^ (Pasteur, 1995; Barnett, 2003). This role is fulfilled by the ADH enzyme that oxidizes acetaldehyde to ethanol, thereby making this the crucial step in this metabolic pathway. Although overexpression of ADH in some plant species has been shown to ameliorate growth and survival under oxygen limitation (Ismond, 2003; Tesniere *et al.*, 2006), this enzymatic activity has been shown to be non-limiting for fermentation. Instead, it was observed that, in different species, it is the preceding decarboxylation of pyruvate to acetaldehyde that constitutes a bottleneck reaction, due to the low catalytic efficiency of PDC (Ismail, 1990; Drew, 1997; Shiao *et al.*, 2002). Here, we report that, in *A. thaliana*, abolition of ADH activity, as displayed by the *adh1* mutant (Figs 6A, 7B), did not impair plant tolerance to anoxic conditions as much as reduction of PDC activity (Fig. 3A, B). Thereby one may infer that pyruvate depletion by PDC is more relevant, under anoxia, than NADH oxidation via ADH to support glycolysis.

This observation apparently seems to challenge the paradigm of ethanol fermentation as the main strategy to enable energy production via glycolysis in plants. Indeed, it has been proposed that fermentation serves an additional purpose for maintaining low pyruvate levels, which otherwise would stimulate respiration and lead to the rapid onset of anoxic conditions when oxygen availability in the surrounding environment is limiting (Zabalza *et al.*, 2009). However, we also observed that mutant Arabidopsis seedlings, whose ADH1 enzyme was genetically inactivated, still produced ethanol under hypoxia, although to a lesser extent than wild-type plants (Fig. 6D), possibly with concomitant NADH oxidation. The ethanol released in the medium by the *adh1* mutant was not significantly lower in double *adh1adh*2 mutants (Fig. 6A), confirming that, despite these two proteins sharing the highest similarity within the Arabidopsis proteome, their function does not overlap and ADH1 is the only ethanol-producing enzyme among Arabidopsis ADHs. Moreover, knocking out the two hypoxia-inducible PDCs also reduced ethanol production to the same extent in seedlings, indicating a similar effect as a consequence of hindering either of the steps of ethanol fermentation (Fig. 6A). Since ethanol release in these mutants occurred only under hypoxic conditions (Fig. 6D), the existence of hypoxia-induced alternative production of ethanol can be hypothesized.

The catalysis involved in this process, however, is expected to possess different biochemical requirements, since ADH activity was completely abolished in the *adh1* mutant (Fig. 6D). Given that ADH2 did not contribute to ethanol production (Fig. 6A), we hypothesize that other more distantly related ADH-like proteins in Arabidopsis are unlikely to be able to perform this metabolic task, since they do not share sequence motifs conserved among plant and animal ethanol-processing ADHs (Fig. 5). To the best of our knowledge, while ethanol catabolism via non-ADH pathways has been reported, no alternative reaction leading to ethanol synthesis has been proposed so far. Ethanol oxidation to acetaldehyde has been shown to occur in peroxisomes of animal cells via catalase in a ‘peroxidative’ reaction using H_2_O_2_ as a co-substrate (Bradford *et al.*, 1993, 1999). An alternative ethanol oxidative pathways relies on the activity of cytochrome P450 (CYP2E1) in microsomes of animal cells (Heit *et al.*, 2013). However, the existence of these pathways has not been confirmed to operate in plant cells. In the light of the expanding information about endophytic bacteria associated with plant cells (Berg *et al.*, 2016), it is tempting to speculate that the additional production of ethanol observed in our experimental conditions might actually derive from bacteria that resides inside plant tissues or cells. On the other hand, the inability of the closest homologue of ADH1 in *M. polymorpha* to complement the *adh1* mutation in Arabidopsis (Fig. 7), and the substantial production of ethanol in this ancient bryophyte (Fig. 1), support the idea of an alternative, as yet unknown, plant enzyme able to support ethanol production under hypoxia.

The ADH-like sequences identified in bryophytes and tracheophytes are likely to have originated as differentiation of class III ADHs, which arose early in evolution as a solution to the need for endogenous as well as exogenous formaldehyde detoxification (Danielsson and Jörnvall, 1992; Strommer, 2011). This class of enzymes is now believed to play a major role in nitric oxide signalling in plants (Hong *et al.*, 2008; Frungillo *et al.*, 2014), which can also explain the severe reduction in hypoxic germination observed for the *adh2/gsnor* mutant (Fig. 4C). In agreement with this perspective of ADH evolution, our comparison of land plant ADH-like sequences led to reliable clustering of GSNOR-like sequences with at least one member for each species considered, whereas less conserved grouping was generated for ADH1-like sequences in primitive land plants (Fig. 2), suggesting a potential different role for these sequences.

Ethanol fermentation in yeast is considered as an adaptive strategy to outcompete other microbial communities for substrate utilization (Dashko *et al.*, 2014; Hagman and Piškur, 2015), whereas in animals the opposite reaction has been proposed to have arisen as a means to detoxify the high ethanol content of rotting fruits (Hernández-Tobías *et al.*, 2011). Instead, the reason behind the evolution of ethanol fermentation in plants has been related to its metabolic importance in cells of sessile organisms under anaerobic conditions (Dolferus *et al.*, 1997), as discussed above. Exploitation of this pathway has later expanded in angiosperms as an ancillary process during the ripening syndrome of fruits and potentially attracts frugivorous animals responsible for seed dispersal (Dudley, 2004). If this notion holds true, it can be hypothesized that plants may have developed alternative pathways of ethanol production in order to assist ADH-based catalysis under oxygen-limited conditions. Forward genetics approaches may be used in the future to test this hypothesis. Genetic reporters for ethanol production, such as the AlcA/AlcR module (Roslan *et al.*, 2001) and the *gek1* mutant, which is ethanol hypersensitive (Hirayama *et al.*, 2004), may be especially useful for this purpose.

Our study serendipitously revealed a new regulatory step imposed over ADH activity in plants, at the post-transcriptional level. In fact, we measured higher ADH protein amounts, and thereby activity, in *pdc1pdc2* mutant seedlings. We speculate that this may be induced by pyruvate accumulation in the mutant, although its aerobic consumption via PDH and AlaAT should not allow it (Fukao and Bailey-Serres, 2004). Indeed, Zabalza *et al.* (2009) reported enhanced ADH activity in response to pyruvate supplementation in pea roots. Moreover, it is possible that this increase is restricted to tissues characterized by low oxygen availability under perfectly aerobic conditions (van Dongen and Licausi, 2015), or rather that a signal originates from these hypoxic niches to induce ADH accumulation elsewhere. *ADH1* mRNA has been shown to be cell to cell mobile via the phloem (Thieme *et al.*, 2015). Since *ADH1* mRNA levels were not affected in the *pdc1pdc2* mutant (Fig. 6B), this regulatory step is expected to occur via either enhanced translation or protein stabilization, and be unrelated to the actual oxygen levels. The 5'-untranslated regions (5'UTRs) *of N. tabacum* and *Z. mays* ADH have been shown to act as potent translational enhancers, although potential metabolite regulation of such activity was not tested (Satoh *et al.*, 2004; Mardanova *et al.*, 2008). At the post-translational level, the hypoxia-inducible ADH of maize, together with other anaerobic polypeptides, has been shown to be highly stable under anoxia by pulse–chase experiments, although, also in this case, this was not assessed under aerobic conditions (Sachs *et al.*, 1980). Additional experiments using proteolysis inhibitors to test whether ADH1 protein levels increase might shed new light on this regulatory pathway.

### Concluding remarks

**Fig. 8.**
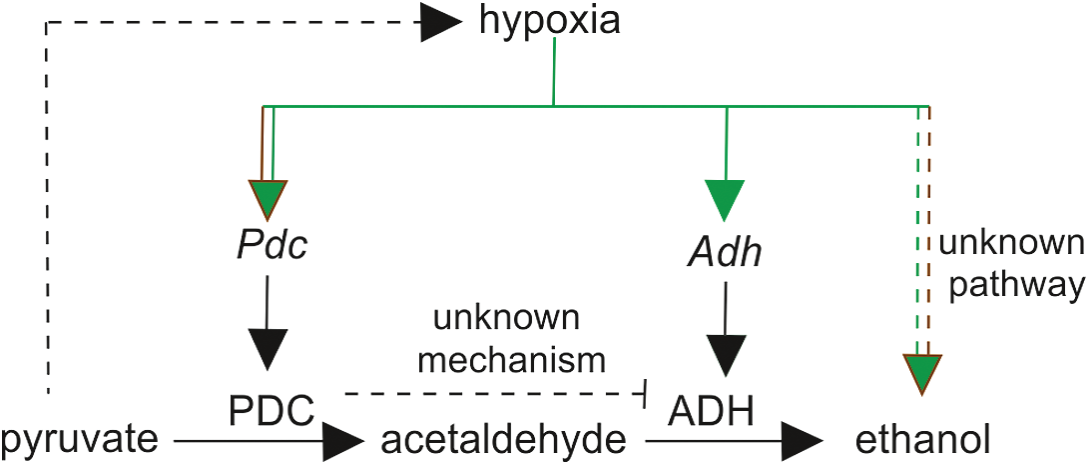
Schematic summary of the hypoxic regulation of ethanol fermentation in plants. Hypoxia induces the expression of genes (italics, lower case) coding for enzymes (upper case) responsible for pyruvate decarboxylation (pyruvate decarboxylase, PDC) and acetaldehyde reduction to ethanol (ADH, alcohol dehydrogenase). Pyruvate accumulation stimulates mitochondrial oxygen consumption through respiration (Zabalza *et al.*, 2009). In addition to ADH and PDC induction, an as yet unknown mechanism contributes to anaerobic ethanol production. Inactivation of PDC activity fosters ADH protein accumulation, and thereby activity. Parallel brown and green lines indicate conservation of regulation across lower and higher plants. Green lines describe control steps identified exclusively in higher plants.

In summary, our study addressed the relevance of ethanol fermentation from both a physiological and evolutionary perspective. The results obtained confirm that ethanol production is a conserved feature in land plants and suggest that additional enzymatic catalysis may be required to support this reaction, independently of the known ethanol-processing ADH identified in seed plants (Fig. 8). Additionally, we reveal the occurrence of a novel regulatory step for ADH activity at the post-transcription level (Fig. 8). Despite the overall generation of questions rather than answers, we believe that these results will stimulate the investigation of ethanol fermentation in plants, aimed at the improvement of crop adaptation to oxygen limitation.

## Supplementary data

Supplementary data are available at *JXB* online.

Fig. S1. Gas chromatographic identification of ethanol in growth medium where no plant was grown (blank), 96% (v/v) ethanol standard, and MS medium where wild-type and *adh1* plants were incubated for 12h in hypoxia (1% O_2_ v/v).

Dataset S1. Multialignment of plant ADH-like protein sequences

Dataset S2. Multialignment of plant and animal ADH/GSNOR-like proteins.

Table S1. List of oligonucleotides used as qPCR primers.

Table S2. List of ADH and PDC homologous sequences used in this study.

Table S3. Expression of ADH- and PDC-like genes in plant species belonging to different phyla.

Table S4. List of ADH-like sequences in the *Arabidopsis thaliana* genome.

Supplementary Material

## Abbreviations:

ADH: alcohol dehydrogenase
CAD: cinnamyl alcohol dehydrogenase
ERF: ethylene responsive factor
GSNO: nitrosylated glutathione
GSNOR: nitrosylated glutathione reductase
LDH: lactate dehydrogenase
PDC: pyruvate decarboxylase
TCA: tricarboxylic acid
