# Supplementary material for: Conservation of ethanol fermentation and its regulation in land plants
Source: J Exp Bot. 2019 Feb 28;70(6):1815–27. doi: 10.1093/jxb/erz052 (PMC6436157; doi:10.1093/jxb/erz052)
Supplement: Supplementary Material [file erz052_suppl_supplementary_material.zip › erz052_suppl_jexbot232983-file001.pdf]

Supporting Information for submission

Conservation of ethanol fermentation and its regulation in land plants

Liem T. Bui, Giacomo Novi, Lara Lombardi, Cristina Iannuzzi, Jacopo Rossi, Antonietta Santaniello, Anna Mensuali, Francoise Corbineau, Beatrice Giuntoli, Pierdomenico Perata, Mirko Zaffagnini, Francesco Licausi

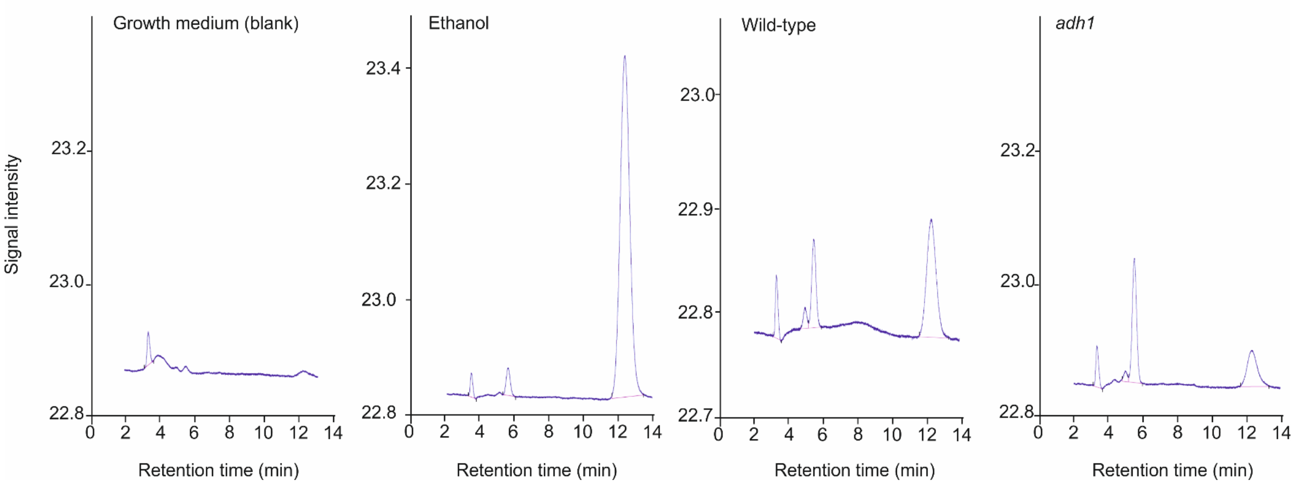

Fig S1. Gas chromatograph identification of ethanol in growth medium where no plant was grown (blank), 96% (v/v) ethanol standard and MS media where wild-type and *adh1* plants were incubated for 12h in hypoxia (1% O_2 v/v).

Table S1. List of oligonucleotides used as PCR and qPCR primers

**Gene name**	**Primer Fw (5'→3')**	**Primer Rv (5'→3')**
Pinus pinea Ubiquitin1	caatttcagggacgagagga	gaaggagcagtggagtcctg
Pinus pinea ADH2	gactgcagcatcgaatgcac	agtcctgccatgatggttgg
Pinus pinea ADH1	cgagtgcacagggaatgtca	gtgtccctcattcagacgc
Pinus pinea ADH3	ttggacatgaagcagctgga	tttactggagaatgcgggga
Pinus pinea PDC1	gtgttggtaggtgggccaaa	tatcccgtggctgtaatgcc
Pteris vittata Act1	caacaggtatcgtgctcgactct	ggcaatgcgtaaccctcataa
Pteris vittata Histone3.2	gggtttacattcagcgaagc	gctttccctccagtggactt
Pteris vittata 18SrRNA	ggttgggggcattcgtattt	tggcaaatgctttcgcagta
Pteris vittata GSNOR-like	ctgggtgatcggaaggtgag	tgttgggtcgtcattttccc
Pteris vittata ADH1-like	tcggccaaagttctgacttga	tcaggtgcttcaatgcacaaa
Pteris vittata PDC1	acctgcatttggatgcgtct	acctgcatttggatgcgtct
Selaginella moellendorffii 6PGD	gctcatggatcccgagtttg	tccactcgtcgtaggtg
Selaginella moellendorffii CLATH	gtcccaatgttcactgcctc	tcttttcccacaccttgagaaac
Selaginella moellendorffii PDF1	actgagactttcggcggtag	cacgccaggacttatcctgag
Selaginella moellendorffii ADH1-like1	agctgtggcatgacaacaggac	gagaagggatcgacagtggcaa
Selaginella moellendorffii GSNOR-like1	ggagcaaccgagtttgttaatccg	atctgtgagatcgaccagcacctg
Selaginella moellendorffii ADH1-like2	catgacaaaccagtgcaagaggtg	attcccaacgcactcgatggtg
Selaginella moellendorffii PDC1	cctcttcaagcacatccaggag	gtgcagtgattgctgagactgg
Selaginella moellendorffii PDC2	ttggtgatggaagtttccaggtg	caaccatgatcaggcaagagca
Physcomitrella patens Tub2	gactgcttgcaaggtttccaac	gttcaagtcgccaaacgaagga
Physcomitrella patens ADH1-like	agacaagcctgttgaggaggtc	gcgtttgagtgtgtaggcaatcc
Physcomitrella patens PDC4	tggtgatggaagtttccaggtgac	ggtccatcgtggatctcaacttcg
Marchania polymorpha Act1	gagcgcggttactctttcac	gaccgtcaggaagctcgtag
Marchania polymorpha CDPK	aagaatgcgctcaatcccaacg	tccgaacatcctctacgtcatc
Marchania polymorpha 18SrRNA	ctccctctccggaatcgaac	cttagcgctggcgatgtttc
Marchania polymorpha ADH1-like	gtggagcagcagtgtgtcgt	cttagctgcggggctggtac
Marchania polymorpha GSNOR-like	tcgcgcatcaatgaagcctttg	tgctgtaatccagcgcaatccg
Marchania polymorpha PDC1	cgacgggtgcttgttcgagt	acccgcttgtccttggaagc
Arabidopsis thaliana UBQ10	ggccttgtataatccctgatgaataag	aaagagataacaggaacggaaa catagt
Arabidopsis thaliana ADH1	tattcgatgcaaagctgctgtg	cgaacttcgtgtttctgcggt
Arabidopsis thaliana PDC1	cacagaatcttcaatgttcttacc	ccatgataaagcgtacatggaa
Arabidpsis thaliana PDC2	ccccaaatccgcagtagagt	cctcaaggggacacacattt
Salk_LBb1	gcgtggaccgcttgctgcaact	
Sail_LB1	gccttttcagaaatggataaatagcct tgcttcc	
At4g33070 (AtPDC1)	cggtggtgaacaatttagatga	atctcagagcaaaaaggagtgc
At5g54960 (AtPDC2)	atcgatactgcgatttcaactg	cattcgggttatctctcaaagg

Table S3. Relative mRNA level of genes coding for ADH- and PDC-like proteins in *Pinus pinea, Pteris vittata, Selaginella moellendorffii, Physcomitrella patens,* and *Marchantia polymorpha*. Plants were subjected to hypoxia (1% O_2 v/v), or maintained under normoxic conditions, at ZT0 and samples were collected at intervals of four hours. mRNA quantification was peformed by means of realtime RT qPCR using primers specified in Supplementary Table Sy. The values are shown as average (AVG) and standard deviation (SD) of four independent biological replicates. Statistical significance of differences between normoxic and hypoxic values was assessed for each zeitgeber time (ZT) point applying a T-test.

	**Normoxia**		**Hypoxia**		
Arabidopsis thaliana					
ADH1	**AVG**	**SD**	**AVG**	**SD**	**P-value**
ZT0	1	0.40242			
ZT4	1.032866	0.324911	3.488056	1.247579	0.01
ZT8	6.998732	1.638936	15.50835	2.450622	<0.001
ZT12	8.081166	4.373322	19.58421	5.367431	0.02
PDC1	**AVG**	**SD**	**AVG**	**SD**	**P-value**
ZT0	1	0.109677			
ZT4	0.339037	0.14105	43.13436	7.802342	<0.001
ZT8	1.173882	0.200198	70.94706	37.88933	0.01
ZT12	0.693069	0.324354	90.649	23.36243	<0.001
PDC2	**AVG**	**SD**	**AVG**	**SD**	**P-value**
ZT0	1	0.891655			
ZT4	0.039248	0.032211	37.50102	15.28467	0.00
ZT8	0.2908	0.171177	26.17281	20.13816	0.04
ZT12	0.138958	0.138892	49.25639	18.81004	0.00
Pinus Pinea					
ADH2	AVG	SD	AVG	SD	P-value
ZT0	1.00	0.43			
ZT4	1.31	0.95	1.00	0.52	ns
ZT8	1.28	0.29	1.42	0.43	ns
ZT12	1.27	0.51	2.12	0.14	ns
ADH1	AVG	SD	AVG	SD	
ZT0	1.00	0.61			
ZT4	3.12	1.70	76.93	50.12	0.03
ZT8	0.80	0.66	137.22	91.84	0.25
ZT12	0.88	0.08	332.59	20.01	<0.001
ADH3	AVG	SD	AVG	SD	
ZT0	1.00	0.37			
ZT4	1.10	0.59	2.98	1.61	ns
ZT8	1.00	0.16	6.50	4.33	0.04
ZT12	0.87	0.15	14.52	5.49	0.00

PDC1	AVG	SD	AVG	SD	
ZT0	1.00	0.213498			
ZT4	1.31	0.095304	4.60	1.809193	0.01
ZT8	0.83	0.598533	8.06	1.167337	<0.001
ZT12	0.90	0.267578	15.63	7.083069	0.01

Pteris vittata

ADH1-like	AVG	SD	AVG	SD	
ZT0	1.00	0.24			
ZT4	1.36	0.21	2.13	0.13	<0.001
ZT8	1.86	0.12	2.28	0.30	0.04
ZT12	2.95	0.99	2.83	1.51	ns

PDC1	AVG	SD	AVG	SD	
ZT0	1.00	0.69			
ZT4	1.74	0.75	1.98	0.87	ns
ZT8	1.68	0.29	3.83	0.87	0.00
ZT12	3.41	2.94	0.97	0.28	ns

Selaginella moellendorffii

ADH-like1	AVG	SD	AVG	SD	
ZT0	1.00	0.61			
ZT4	1.91	1.06	7.98	4.45	0.04
ZT8	4.07	2.64	1.60	1.43	ns
ZT12	5.16	1.15	5.86	3.92	ns

GSNOR-like1	AVG	SD	AVG	SD	
ZT0	1.00	0.50			
ZT4	0.43	0.30	0.24	0.30	ns
ZT8	0.60	0.62	0.40	0.24	ns
ZT12	0.96	0.91	0.22	0.06	ns

ADH-like2	AVG	SD	AVG	SD	
ZT0	1.00	0.83			
ZT4	0.82	0.51	0.64	0.72	ns
ZT8	1.17	1.33	1.48	1.10	ns
ZT12	0.17	0.13	0.44	0.36	ns

PDC1	AVG	SD	AVG	SD	
ZT0	1.00	1.02			
ZT4	0.60	0.23	0.91	0.81	ns
ZT8	1.24	1.37	0.26	0.13	ns
ZT12	4.47	2.43	5.22	2.08	ns

PDC2	AVG	SD	AVG	SD	
ZT0	1.00	0.67			
ZT4	1.38	0.69	2.58	1.49	ns
ZT8	1.34	0.51	3.28	1.23	0.03

ZT12	1.17	1.08	0.53	0.39	ns
Physcomitrella patens					
ADH-like	AVG	SD	AVG	SD	
ZT0	1.00	0.17			
ZT4	0.77	0.35	0.66	0.30	ns
ZT8	1.11	0.43	0.82	0.27	ns
ZT12	1.62	0.72	0.78	0.32	ns
PDC1	AVG	SD	AVG	SD	
ZT0	1.00	0.06			
ZT4	1.31	0.27	2.92	1.06	ns
ZT8	0.70	0.34	9.61	4.56	0.01
ZT12	1.57	0.38	4.13	0.70	<0.001
Marchantia polymorpha					
ADH-like					
ZT0	1.00	0.57			
ZT4	0.95	0.39	0.46	0.12	ns
ZT8	0.82	0.24	0.30	0.12	0.01
ZT12	0.52	0.23	0.19	0.02	0.031
PDC1					
ZT0	1.00	0.65			
ZT4	0.78	0.23	4.44	1.94	0.01
ZT8	1.51	0.75	1.56	0.41	ns
ZT12	1.35	0.72	1.66	0.69	ns

Table S4. List of ADH-like sequences identified among proteins encoded in the *Arabidopsis thaliana* genome using AT_ADH1 (At1g77120) as a bait with BLAST (Altshul et al. 1990).

Isoform	Gene model	Name	Score (bits)	E-value
AT1G77120.1	AT1G77120	ADH1	746	0
AT5G43940.1	AT5G43940	ADH2/GSNOR	444	e-125
AT5G43940.2	AT5G43940	ADH2/GSNOR	434	e-122
AT5G24760.1	AT5G24760	no name	404	e-112
AT5G24760.3	AT5G24760	no name	374	e-104
AT5G24760.2	AT5G24760	no name	374	e-104
AT1G32780.1	AT1G32780	no name	372	e-103
AT1G64710.1	AT1G64710	no name	370	e-103
AT1G64710.2	AT1G64710	no name	337	1.00E-92
AT5G42250.1	AT5G42250	no name	327	9.00E-90
AT1G22440.1	AT1G22440	no name	324	1.00E-88
AT4G22110.2	AT4G22110	no name	320	2.00E-87
AT4G22110.1	AT4G22110	no name	320	2.00E-87
AT1G22430.2	AT1G22430	no name	315	7.00E-86
AT1G22430.1	AT1G22430	no name	315	7.00E-86
AT1G64710.3	AT1G64710	no name	276	2.00E-74
AT5G24760.4	AT5G24760	no name	255	4.00E-68
AT1G22430.3	AT1G22430	no name	244	1.00E-64
AT4G22110.3	AT4G22110	no name	219	4.00E-57
AT1G22430.5	AT1G22430	no name	218	8.00E-57
AT1G22430.4	AT1G22430	no name	218	8.00E-57
